# Update on Anticytokine Treatment for Asthma

**DOI:** 10.1155/2013/104315

**Published:** 2013-06-18

**Authors:** Luca Gallelli, Maria Teresa Busceti, Alessandro Vatrella, Rosario Maselli, Girolamo Pelaia

**Affiliations:** ^1^Clinical Pharmacology Unit, Department of Health Science, University “Magna Græcia” of Catanzaro, Campus Universitario “S. Venuta”, Viale Europa-Località Germaneto, 88100 Catanzaro, Italy; ^2^Department of Medical and Surgical Sciences, University “Magna Græcia” of Catanzaro, Campus Universitario “S. Venuta”, Viale Europa-Località Germaneto, 88100 Catanzaro, Italy; ^3^Department of Respiratory Medicine, University of Salerno, 84084 Fisciano (SA), Italy

## Abstract

Current advances in the knowledge of asthma pathobiology suggest that anticytokine therapies can be potentially useful for the treatment of this complex and heterogeneous airway disease. Recent evidence is accumulating in support of the efficacy of anti-IL-4, anti-IL-5, and anti-IL-13 drugs. Therefore, these new developments are now changing the global scenario of antiasthma therapies, especially with regard to more severe disease. Current findings referring to variability of individual therapeutic responses highlight that the different asthma subtypes need to be well characterized, in order to implement phenotype-targeted treatments which in the near future will hopefully be mainly based on cytokine-directed biologics.

## 1. Introduction

Asthma is a chronic disease of the airways, characterized by inflammatory, structural, and functional changes responsible for bronchial hyperresponsiveness and usually reversible airflow limitation [1, 2]. It constitutes a heavy medical, social, and economic burden because its prevalence is continuously increasing worldwide [3]. Indeed, asthma affects over 300 million people around the world, and some epidemiologic projections estimate that such a number will further increase during the next decades [4]. Although a good control of asthma symptoms can be achieved in a vast majority of patients by current standard therapies mainly based on combinations of inhaled corticosteroids and *β*_2_-adrenoceptor agonists, eventually associated with oral leukotriene inhibitors [5, 6], a small percentage (about 5–10%) of asthmatic subjects who are affected by the most severe subtypes of the disease, though receiving the best available conventional treatments remain symptomatic and inadequately controlled, thus having a poor quality of life. In these patients, asthma symptoms can be further worsened by concomitant comorbidities including rhinitis, sinusitis, gastrooesophageal reflux, obesity and obstructive sleep apnoea [7–10]. Patients with uncontrolled asthma exhibit a high risk of serious morbidity and mortality, thereby representing the most severe sector of the overall phenotypic asthma spectrum, and thus being characterized by the greatest unmet medical needs [11, 12]. Therefore, though being a minority of the global asthmatic population, patients with severe asthma are those who use the largest share of economic resources and health care services, including emergency visits, hospitalizations, and additional consumption of drugs utilized for recurrent exacerbations. A further social and economic impact of difficult-to-treat asthma arises from the frequent loss of school and work days, due to such a disabling condition. Moreover, patients with severe asthma often show a tendency to anxiety and depression, which can further impair disease control by reducing their compliance to prescribed medications. Therefore, for the poorly controlled asthma phenotypes, additional therapeutic approaches are absolutely required.

In this regard, a large body of evidence also suggests that many cytokines released by both immune-inflammatory and airway structural cells significantly contribute to shape the several different disease phenotypes [13–15]. Indeed, basic and clinical research has identified several potentially suitable cytokine targets for antiasthma therapies [16, 17]. Such considerations highlight the potential importance of biologic treatments directed against proinflammatory cytokines, including monoclonal antibodies and small-molecule inhibitors. In particular, biologics may represent useful adjunctive therapies, especially for patients with more severe asthma which is not fully responsive to conventional treatments alone [18–20]. Variable responses have been observed using experimental cytokine-directed therapies, probably because of the significant differences occurring among distinct asthma phenotypes. This implies that biologic drugs need to be addressed against the molecular targets which are relevant to each phenotypic subgroup of asthma. In this regard, the aim of the present review is to outline, after recalling the most recent advances in asthma pathobiology, the newly developing anticytokine therapies for asthma.

## 2. Pathobiology of Asthma

Asthma is a complex and heterogeneous disease characterized by various pathologic and clinical phenotypes, based on different patterns of airway inflammation involving immune-inflammatory cell types such as T and B lymphocytes, mast cells, eosinophils, basophils, neutrophils, and dendritic cells, as well as structural cellular elements including both epithelial and mesenchymal cells. This widespread respiratory disease, which originates from multiple interactions between genetic factors and environmental agents such as allergens, respiratory viruses, and airborne pollutants, is characterized by recurrent episodes of dyspnoea, wheezing, chest tightness, and cough, usually associated with a reversible airflow limitation and an exaggerated bronchoconstrictive response to several different stimuli (airway hyperresponsiveness). Since many decades ago, asthma has been classified into two major phenotypes known as allergic (atopic) or “extrinsic” asthma and “intrinsic” (nonatopic) asthma [21]. Atopic asthma is the dominant disease manifestation during early life and young adulthood, whereas the nonatopic form is more frequent in older patients, thus often characterizing the so-called late-onset subtype of asthma.

The main pathologic feature of asthma is chronic inflammation, frequently associated with structural changes of airway wall, collectively defined as tissue remodelling [22]. Atopic asthma is widely believed to be triggered by an immune-inflammatory response driven by T-helper type 2 (Th2) lymphocytes. This so-called “Th2-high” subphenotype of asthma arises from a complex interplay between the innate, and adaptive branches of immune system [23, 24]. In particular, aeroallergens that cause atopic asthma including pollens, house dust mite and animal dander, often have proteolytic properties and also contain trace amounts of bacterial constituents such as lipopolysaccharide (LPS) [25]. By virtue of these features, once penetrated into airway epithelium inhaled allergens can activate the Toll-like receptor (TLR) class of pattern recognition receptors involved in innate immunity. TLR activation induces the synthesis of innate cytokines such as thymic stromal lymphopoietin (TSLP) and interleukins-25 (IL-25) and -33 (IL-33), able to elicit the development of Th2 adaptive responses. Furthermore, TLR stimulation also promotes the epithelial release of C-C chemokine ligands 2 (CCL2) and 20 (CCL20), which favour the recruitment and maturation of dendritic cells [15]. The latter extend their intraepithelial processes into the airway lumen and capture aeroallergens. This uptake of inhaled antigens is stimulated by IgE bound to high affinity receptors (Fc*ε*RI) located on the surface of dendritic cells. Interaction of IgE with Fc*ε*RI receptors expressed by dendritic cells facilitates allergen internalization inside their cytoplasm [26], where antigens are processed by cathepsin S whose action, thus, results in the generation of allergenic peptide fragments. The latter are then loaded within the context of HLA molecules belonging to the class II of the major histocompatibility complex (MHC class II) expressed by dendritic cells, that migrate to T-cell areas of regional thoracic lymph nodes where antigen presentation to T lymphocytes takes place. Recognition of specific antigenic peptides by T-cell receptors triggers sensitization and the following adaptive immune response. Allergen-dependent activation of naïve T lymphocytes requires the interaction of their costimulatory molecules (CD28, ICOS, and OX40) with the respective counter-ligands expressed by dendritic cells (CD80/B7.1, CD86/B7.2, ICOS ligand, and OX40 ligand) [27]. The type of antigen presentation-dependent differentiation of T lymphocytes is critically determined by the cytokine milieu. In particular, IL-12 produced by dendritic cells promotes Th1 polarization whereas commitment towards the Th2 lineage is driven by IL-4, probably released from mast cells, basophils, eosinophils, and T cells [28]. Moreover, TSLP is secreted in large amounts by bronchial epithelial cells and mast cells of asthmatic patients, thus inducing dendritic cells to release CCL17 and CCL22 chemokines, that recruit Th2 cells upon binding to their CCR4 receptor [29]. Activated Th2 lymphocytes then leave lymph nodes and enter the airways, where further allergen deposition and antigen presentation by local dendritic cells occur. As a consequence, Th2 cells expressing the CCR4 chemokine receptor synthesize large amounts of cytokines encoded by the gene cluster located on the long arm of chromosome 5, including IL-3, IL-4, IL-5, IL-6, IL-9, IL-13, and granulocyte macrophage colony stimulating factor (GM-CSF). These cytokines and growth factors stimulate maturation and recruitment of other immune cells involved in the allergic cascade, such as eosinophils and mast cells [30]. In particular, eosinophil differentiation in the bone marrow is promoted by IL-5, whose action is synergized by eosinophil-recruiting chemokines like eotaxin, released by both inflammatory and airway resident cells [31]. IL-4 and IL-13 act on B lymphocytes by driving immunoglobulin class switching towards the production of IgE [12]. IL-9, secreted by a further subset of T lymphocytes (Th9) derived from Th2 cells, attracts mast cells and triggers their differentiation [32]. 

Whereas Th2 lymphocytes are mainly involved in the development of an inflammatory phenotype referred to as eosinophilic asthma, other Th cell subsets induce airway neutrophilic inflammation, often associated with the most severe clinical phenotypes. In particular, a specific lineage of CD4^+^ effector T lymphocytes, expressing IL-17 and thus named Th17, appears to play a pivotal role in airway neutrophilia [33, 34]. Indeed, in lung tissue sections from asthmatic patients there is an overexpression of IL-17A and IL-17F, whose levels correlate with asthma severity, especially in subjects with neutrophilic, steroid-resistant disease [35]. In mice, Th17 lymphocyte differentiation from uncommitted cell precursors requires IL-6 and transforming growth factor-β (TGF-β), and IL-17 expression is further enhanced by IL-23 [36]. IL-17A and/or IL-17F stimulate airway structural cellular elements, like bronchial epithelial cells and subepithelial fibroblasts, to secrete powerful neutrophil chemoattractants such as IL-8 and CXCL1/GRO-*α* [36]. Th17 cells may contribute to the pathogenesis of allergic asthma, thus worsening its severity [37]. Therefore, it is reasonable to speculate that a predominantly Th2-mediated airway eosinophilia is likely responsible for mild and moderate atopic asthma, whereas concomitant activation of both Th2 and Th17 cells can be frequently associated with a mixed eosinophilic/neutrophilic inflammatory phenotype underlying more severe disease.

Another cytokine that is implicated in the pathogenesis of severe neutrophilic asthma is tumour necrosis factor-*α* (TNF-*α*), produced by CD4^+^ T lymphocytes, monocytes and/or macrophages as well as several other cell types, which exerts pleiotropic effects on inflammatory and structural airway cells [38, 39]. Combined patterns of both neutrophilic and eosinophilic airway infiltrates may occur in recurrent acute relapses of asthma, that characterize the so-called exacerbation-prone asthmatic phenotype [40]. These exacerbations can be caused by allergens and especially by respiratory viruses, whose pathogenic effects within the airways of asthmatic patients are favoured by a deficient epithelial synthesis of antiviral cytokines such as interferons β (IFN-β) and *λ* (IFN-*λ*) [41].

Across the wide severity spectrum of allergic asthma phenotypes, defective numbers and/or functions of specific regulatory T lymphocytes (T_reg_ cells) have been detected [42, 43]. Several different T_reg_ lymphocyte subsets have been identified, including naturally occurring CD4^+^CD25^+^ cells expressing the transcription factor FOXP3 (forkhead box P3). T_reg_ cells exert their immune-modulatory functions through direct and indirect mechanisms. In particular, T_reg_ cells produce anti-inflammatory cytokines like IL-10 and TGF-β, express inhibitory factors such as CTLA4 (cytotoxic T lymphocyte antigen 4), and also downregulate MHC class II proteins and CD80/CD86 costimulatory molecules expressed by antigen presenting cells [44]. A defective function of T_reg_ cells is probably also a feature of intrinsic, nonatopic asthma triggered by microbial superantigens. The latter can indeed suppress the immune-modulatory role of T_reg_ cells, thus enhancing the activity of both CD4^+^ and CD8^+^ T lymphocytes [45].

In asthma, chronic inflammation is frequently associated with dynamic structural changes that involve all airway wall layers and extend from proximal, large-to-distal, small airways. Such a tissue remodeling occurs in both atopic and nonatopic asthma [46], and includes epithelial shedding, goblet cell, and mucous gland hyperplasia, enhanced deposition of extracellular matrix proteins leading to subepithelial fibrosis, and increased angiogenesis and hypertrophy/hyperplasia of smooth muscle cells, which acquire a highly proliferative, secretory, and contractile phenotype [47]. It is currently believed that airway remodeling in asthma is largely due to complex interactions between bronchial epithelium and the underlying mesenchyme, resulting from a reactivation of the developmental epithelial-mesenchymal trophic unit (EMTU) responsible for lung morphogenesis during fetal life [48]. Within the context of EMTU reactivation, a crucial role is played by TGF-β [49], a fibrogenic growth factor whose levels are upregulated in asthmatic airways because of an increased release from immune-inflammatory cells, as well as from damaged epithelial cells and activated mesenchymal cells. Other growth factors contributing to airway remodeling in asthma include endothelin-1 (ET-1), epidermal growth factor (EGF), platelet-derived growth factor (PDGF), and vascular endothelial growth factor (VEGF) [50–57]. Overall, airway remodeling results in thickening of bronchial and bronchiolar walls, leading to reduction of airway calibre and fixed airflow limitation that is correlated with a progressive decline of respiratory function.

**Figure 1:**
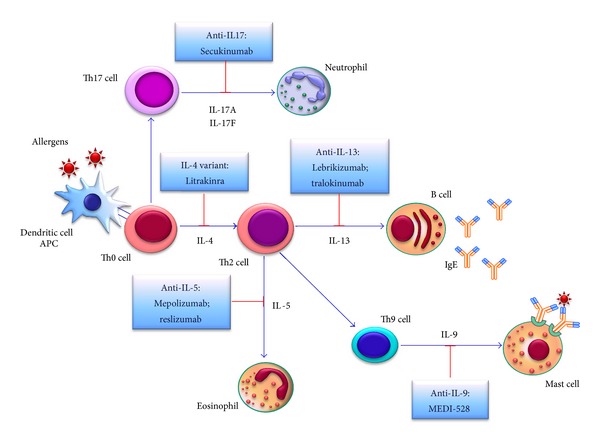
Anticytokine therapies for asthma. The main suitable cytokine targets for developing antiasthma biologics are depicted. See text for further details.

These recent advances in the understanding of asthma pathobiology, and especially a better knowledge of the cellular and molecular mechanisms that underlie uncontrolled asthma, may have important prospective therapeutic implications. In particular, the improved awareness of the inflammatory and immune events involved in cytokine cascades is unravelling potential targets for the development and implementation of new biological therapies. Several different antiasthma biologics are currently under different stages of investigation, including molecules directed against IL-5, IL-4, IL-13, IL-9, GM-CSF, and TNF-*α*. Moreover, IL-17 and IL-23, as well as the innate cytokines TSLP, IL-25, IL-33, and IL-27, are additional interesting targets for future asthma therapies. The main anticytokine strategies under current development for asthma treatment are schematically illustrated in Figure 1. 

## 3. Anti-IL-5

IL-5 plays a crucial role in the growth, maturation, and activation of eosinophils [31]. Therefore, anti-IL5 therapeutic strategies may potentially be effective in the treatment of eosinophilic asthma phenotypes [58]. In this regard, several preclinical studies have been carried out in experimental animal models of asthma. In particular, pretreatment with the anti-IL-5 blocking antibody TRFK-5 was able to inhibit eosinophil influx into the airways of allergen-sensitized mice [59]. Furthermore, TRFK-5 also suppressed airway eosinophilic infiltration and bronchial hyperresponsiveness in a nonhuman primate model of asthma [60]. More recently, other antibodies such as mepolizumab, reslizumab, and benralizumab have been developed [61].

Some clinical trials performed in heterogeneous populations of patients with mild or moderate chronic persistent asthma have shown that mepolizumab, a humanized monoclonal antibody against IL-5, is safe and can effectively reduce eosinophil numbers in airways and blood [62, 63]. However, these effects were not paralleled by significant improvements in asthma symptoms, lung function, and bronchial hyperresponsiveness. More recently, mepolizumab has been tested in selected subtypes of chronic severe asthma, characterized by frequent exacerbations and airway eosinophilia refractory to inhaled and systemic corticosteroid therapies [64, 65]. Taken together, the results of these two small trials demonstrate that mepolizumab was well tolerated during a 12-month treatment period and dramatically decreased asthma exacerbations and eosinophil levels in both blood and induced sputum. Such findings have been further corroborated by the large, multicentre DREAM study, carried out in 621 patients with severe, exacerbation-prone, eosinophilic asthma who were randomly assigned to four groups receiving at 4-week intervals 13 intravenous infusions of placebo or one of three doses of mepolizumab (75 mg, 250 mg, or 750 mg) [66]. At all dosages used, mepolizumab was well tolerated and effectively decreased the frequency of asthma exacerbations, as well as blood and sputum eosinophil counts [66].

In addition to mepolizumab, another interesting anti-IL-5 biologic drug is reslizumab, an IgG4/*κ* humanized monoclonal antibody. When compared to placebo in patients with poorly controlled eosinophilic asthma, reslizumab has been recently shown to significantly decrease sputum eosinophils and improve lung function, as well as inducing a positive trend toward better asthma control [67]. The antiasthma effects of reslizumab were most pronounced in a subgroup of patients characterized by the highest levels of blood and sputum eosinophils, which were associated with the presence of nasal polyposis [67].

Therefore, all such findings further emphasize the importance of accurate phenotype selection, in order to tailor antiasthma treatments targeted to the peculiar biologic and clinical features of the individual disease expressions. These concepts will eventually also apply to the use of benralizumab, an IgG1 monoclonal antibody directed to IL-5 receptor, that in preliminary investigations has been reported to be quite safe and to effectively reduce peripheral blood eosinophils [68]. 

## 4. Anti-IL-4

IL-4 contributes to asthma pathophysiology by inducing Th2 cell differentiation and expansion, isotype switching of B cells to IgE synthesis, as well as eosinophil recruitment, development of mast cells and mucous metaplasia [50]. Moreover, IL-4 is also involved in airway remodeling by upregulating collagen and fibronectin production. Several studies aimed to evaluate the effects of anti-IL-4 therapies in asthma treatment have yielded conflicting results [69]. In murine models of allergen-induced asthma, blockade of either IL-4 or its receptor has been shown to inhibit eosinophil influx into the airways and IL-5 release from T cells, as well as decreasing lung inflammation, serum IgE levels, and airway hyperresponsiveness to methacholine [70, 71]. However, although the humanized anti-IL-4 monoclonal antibody pascolizumab is well tolerated, it lacks clinical efficacy in asthmatic patients [16]. Similarly, despite some promising preliminary findings regarding the soluble recombinant human IL-4 receptor altrakincept, no significant clinical efficacy has been later confirmed [72]. More effective appears to be pitrakinra, a bioengineered variant of IL-4 that acts as an antagonist at the heterodimeric receptor complex (IL-4R*α*/IL-13R*α*1) shared by both IL-4 and IL-13 [73]. In particular, when administered by either subcutaneous or inhaled route, pitrakinra is safe and inhibits allergen-induced early and late asthmatic responses, as well as disease exacerbations in selected phenotypes of eosinophilic asthma [50, 74]. Moreover, in the first large pharmacogenetic, placebo-controlled investigation of the IL-4/IL-13 pathway, three doses (1 mg, 3 mg, or 10 mg twice daily for 12 weeks) of inhaled pitrakinra were tested in patients with moderate-to-severe asthma [75]. Although this trial failed to demonstrate clinical efficacy in the whole study population, at the 10 mg dosage pitrakinra significantly lowered the frequency of asthma exacerbations in individuals carrying specific single nucleotide polymorphisms in the gene encoding IL-4R*α*, located within the 3′ untranslated region (rs8832GG and rs1029489GG genotypes) [75].

More recently, a fully human monoclonal antibody directed against the *α* subunit of the IL-4 receptor (dupilumab) has been tested in patients with persistent, moderate-to-severe asthma and blood or sputum eosinophilia. When compared with placebo, dupilumab induced a significant decrease in asthma exacerbation rate during withdrawal of inhaled therapy with corticosteroids and long-acting *β*_2_-adrenergic agonists, paralleled by a marked improvement of respiratory function and by reduced levels of Th2-associated biomarkers such as eosinophils, exhaled NO, and eotaxin-3 [76]. 

## 5. Anti-IL-13

IL-13 is a key target for the development of new antiasthma therapeutic strategies because of its involvement, together with IL-4, in several different aspects of airway inflammation and remodeling, including mucus production, IgE synthesis, recruitment of eosinophils and basophils, and proliferation of bronchial fibroblasts and airway smooth muscle cells [50]. Anti-IL-13 treatments performed in experimental animal models of chronic allergic asthma can markedly attenuate IgE synthesis, airway eosinophilia, and bronchial structural changes.

In clinical trials, the anti-IL-13 monoclonal antibody anrukinzumab has been able to significantly inhibit allergen-induced late asthmatic responses within 14, but not 35 days after administration [77]. Furthermore, it has also been shown that the anti-IL-13 monoclonal antibody lebrikizumab exerts an effective antiasthma action in the so-called “Th2-high” asthmatic phenotype, characterized by an overexpression of IL-13-inducible genes such as periostin, an extracellular matrix protein [78]. In this study, the overall frequency of adverse events resulted to be similar in the two groups of asthmatic subjects undergoing treatment with lebrikizumab or placebo, respectively, in addition to standard inhaled therapy. With regard to efficacy, lebrikizumab induced a relevant improvement of lung function in patients with moderate-to-severe asthma displaying high serum levels of periostin. In particular, at week 12 the reported percentage increases in forced expiratory volume in one second (FEV_1_), with respect to baseline values, being 5.5% in the whole lebrikizumab-treated group, 8.2% in the high-periostin subgroup, and 1.6% (not significant) in the low-periostin subgroup [78]. This implies that easily detectable biomarkers could be routinely used in clinical practice to identify specific asthmatic phenotypes, characterized by an important pathogenic role of IL-13, and thus being potentially responsive to therapeutic strategies targeted against such a pleiotropic cytokine.

Another humanized anti-IL-13 antibody currently in clinical development is tralokinumab, characterized by favourable pharmacokinetic properties and a good safety profile [79]. Tralokinumab has been recently evaluated in a trial involving 194 adult patients with moderate-to-severe uncontrolled asthma randomly assigned to receive, every 2 weeks through the subcutaneous route in addition to currently available controller therapy, either placebo or one of three different doses (150, 300, and 600 mg) of the IL-13 neutralizing antibody, respectively [80]. Although when compared to placebo tralokinumab did not affect symptom score assessed by the Asthma Control Questionnaire (ACQ), this anti-IL-13 drug reduced the need for rescue medication and significantly improved lung function by eliciting percentage increases in FEV_1_ from baseline ranging from 8.1% (150 mg) to 16.1% (600 mg) [80]. With respect to subjects lacking IL-13 in induced sputum, FEV_1_ changes were far greater in patients having detectable IL-13 sputum levels. Tralokinumab was well tolerated and did not induce any serious adverse event. 

## 6. Anti-IL-9

IL-9 is overexpressed in asthmatic airways, where this cytokine stimulates mast cell proliferation and mucus hyperplasia [81]. In mice, IL-9 blockade reduced airway inflammation and hyperresponsiveness [82]. Moreover, in two randomized phase 2a trials carried out in subjects with mild-to-moderate asthma, the humanized anti-IL-9 monoclonal antibody MEDI-528 exhibited an acceptable safety profile and also evoked a trend toward improvement in asthma symptom scores and disease exacerbation rates [83]. The second of these two clinical studies showed that 50 mg of MEDI-528, administered subcutaneously twice weekly can exert a protective effect against exercise-induced bronchoconstriction [83]. 

## 7. Anti-GM-CSF

GM-CSF is a growth factor overexpressed in asthmatic airways, which plays a key role in eosinophil differentiation and survival [12]. In a murine model of allergic asthma, intranasal administration of a goat anti-mouse GM-CSF polyclonal antibody exerted a significant inhibitory effect on airway inflammation, mucus production, and bronchial hyperresponsiveness [84]. Later, a human anti-GM-CSF monoclonal IgG1 antibody (MT203) has been developed, capable of significantly decreasing survival and activation of peripheral human eosinophils [85]. 

## 8. Anti-TNF-*α*

In murine models of allergen-dependent asthma, the proinflammatory cytokine TNF-*α*, produced by Th1 lymphocytes, macrophages and mast cells, induced airway recruitment of neutrophils and eosinophils via upregulation of epithelial and endothelial adhesion molecules [38]. TNF-*α* is overexpressed in the airways of patients with severe asthma and also directly stimulates airway smooth muscle contraction through changes in intracellular calcium fluxes [86]. Therefore, several drugs targeting TNF-*α* have been evaluated for asthma treatment, including anti-TNF-*α* blocking antibodies such as infliximab and golimumab, as well as the soluble TNF-*α* receptor fusion protein etanercept. Overall, conflicting results have been obtained and serious concerns have been raised with regard to the safety of TNF-*α* blockade, which may cause susceptibility to the development of respiratory infections and human cancers.

Etanercept was preliminarily shown to significantly improve lung function, airway hyperresponsiveness, and quality of life in asthmatic patients expressing high monocyte levels of both TNF-*α* and TNF-*α* receptor [87]. More recently, however, no significant differences between etanercept and placebo have been observed with regard to lung function, airway hyperresponsiveness, quality of life, and exacerbation rate, during a larger randomized trial performed in patients with moderate-to-severe persistent asthma, exhibiting a good drug tolerability [88]. In subjects with moderate asthma, the humanized anti-TNF-*α* monoclonal antibody infliximab was able to reduce the circadian oscillations in peak expiratory flow and the related disease exacerbations [89]. However, a larger study carried out in patients with persistent severe asthma receiving golimumab, another TNF-*α* blocking antibody, did not detect any significant improvement in lung function and disease exacerbations [90]. Moreover, serious adverse infectious and neoplastic events like active tuberculosis, pneumonia, sepsis, and several different malignancies (breast cancer, B-cell lymphoma, metastatic melanoma, cervical carcinoma, renal cell carcinoma, basal cell carcinoma, and colon cancer) were reported. Therefore, the trial was interrupted and it appears to be currently very unlikely that anti-TNF-*α* antibodies will be soon further evaluated the for treatment of severe asthma. However, a subgroup analysis of the patients enrolled in the golimumab trial demonstrated that the drug was beneficial in older patients with late-onset asthma and a history of hospitalizations or emergency hospital visits during the year before screening, who also had lower baseline FEV_1_ levels and a postbronchodilator FEV_1_ increase of >12%. 

## 9. Anti-IL-17 and Anti-IL-23

IL-17A and IL-17F, which are proinflammatory cytokines released by Th17 cells and crucially involved in neutrophilic inflammation as well as in airway remodeling, are significantly upregulated in bronchial biopsies obtained from patients with severe asthma [35]. In this regard, it is noteworthy that in mouse models of allergic asthma an anti-IL-17 antibody lowered the numbers of neutrophils, eosinophils, and lymphocytes detected in bronchoalveolar lavage fluid [91]. Ongoing phase II clinical trials are currently evaluating the efficacy and safety of a fully human IL-17A-specific monoclonal antibody (secukinumab), as well as of a human IL-17-receptor-specific monoclonal antibody (brodalumab), in patients with severe asthma that is not adequately controlled by inhaled corticosteroids and long-acting *β*_2_-adrenergic receptor agonists [16].

Another potential therapeutic approach can be provided by the use of antibodies directed against the IL-17 regulating cytokine IL-23, whose blockade resulted in a significant inhibition of antigen-dependent recruitment of neutrophils, eosinophils, and lymphocytes into the airways of sensitized mice [92]. However, these experimental strategies should be considered with extreme caution because IL-17 is also involved in immune protection against infectious and carcinogenic agents [93]; hence, inactivation of this cytokine could result in an increased risk of opportunistic infections and cancer development.

## 10. Anti-IL-25, Anti-IL-33, and Anti-TSLP

IL-25, IL-33, and TSLP, mainly released from airway epithelium, are overexpressed in asthmatic airways and play a crucial role in driving and stimulating Th2-mediated immune-inflammatory responses [22]. Therefore, these cytokines are currently believed to be suitable targets for novel antiasthma therapies. In mice, an anti-IL-25 monoclonal antibody has been reported to suppress Th2-dependent allergic airway inflammation [94]. Moreover, murine allergic inflammation and airway hyperresponsiveness can be inhibited by antibodies directed against the IL-33 receptor [95]. These antibodies can also markedly inhibit IL-17F expression in human bronchial epithelial cells [96]. A relevant attenuation of mouse allergic airway inflammation has also been observed as a consequence of antibody-induced neutralization of the TSLP receptor [97].

## 11. Anti-IL-27

IL-27 is a monocyte- and macrophage-derived innate cytokine that is probably involved in the pathogenesis of severe, corticosteroid-resistant asthma. Indeed, IL-27 levels are increased in the airways of patients with severe neutrophilic asthma [98]. Moreover, in mouse lung macrophages, IL-27 inhibited nuclear translocation of glucocorticoid receptors [98], which is an essential cellular event for the biological and pharmacological effects of corticosteroids. Therefore, IL-27 may represent a potential target for new therapeutic strategies aimed to provide a better control of severe, steroid-refractory asthma. 

## 12. Potential Problems Related to Anticytokine Therapies

Most anticytokine strategies under current development for the treatment of asthma mainly target Th2-derived cytokines, including IL-5, IL-4, and IL-13, crucially involved in the pathobiology of allergic phenotypes. Therefore, these anticytokine approaches may be beneficial in the management of atopic asthma and other allergic diseases such as allergic rhinitis and atopic dermatitis. Indeed, by significantly reducing eosinophilic tissue infiltration, Th2 cytokine antagonists interfere with a key cellular component of allergic inflammation. However, both patients and physicians should be aware of the time required for anti-Th2 cytokine treatments to alleviate asthma and allergy symptoms, usually ranging from one to a few weeks; this time is necessary to achieve a significant inhibition of the cellular and molecular mechanisms implicated in allergy pathophysiology. On the contrary, some concerns can arise from the consideration that anti-Th2 cytokine therapies can be potentially detrimental in patients with autoimmune disorders. Indeed, it has been recently suggested that IL-4, IL-5, and eosinophils can play a suppressive role in autoimmune mouse models of multiple sclerosis [99]. Thus, the possibility of administering anti-Th2 cytokine therapies to subjects with autoimmune diseases should be considered with extreme caution. Because of the widespread convincement that eosinophils exert a protective action against parasitic diseases, traditional medical judgement could lead to exclude patients with these infections from receiving Th2 cytokine-targeted treatments. However, this mental approach should probably be reconsidered in view of recent studies suggesting that eosinophils can be involved in parasite survival, rather than in parasite killing [100, 101]. Overall, anti-Th2 cytokine therapies are safe and well tolerated, and their main side effects include minor events such as mild injection site reactions, nasopharyngitis, nausea, and headache. Hypersensitivity responses including oedema, papular rashes, and urticaria can rarely occur during treatment with biologic drugs targeting Th2 cytokines; however, these unwanted effects are usually not severe and often resolve after immediate discontinuation of the study drug and nonurgent symptomatic treatment with corticosteroids and antihistamines. 

Different from Th2 cytokine-targeted treatments, much more important problems can arise in asthmatic patients from the use of drugs directed against Th1 cytokines such as TNF-*α*. In particular, the occurrence of dramatic adverse events including serious infections and neoplastic disorders, observed during treatment with golimumab [90], currently discourages the further development of anti-TNF-*α* strategies for asthma therapy. A very careful attention should also be paid to the recruitment of asthmatic patients for clinical studies evaluating the effects of antibodies targeting IL-17A and IL-17F, given the physiologic role of these cytokines in the immunological surveillance against infections and cancer; of course, patients with immunodeficiencies must be excluded from such trials.

## 13. Conclusions

During the last years, both basic and clinical research strategies have identified many attractive molecular targets for asthma treatment. In particular, anticytokine therapies, added to conventional treatments and eventually used in various combinations, according to patient's individual requirements, could lead to significant improvements in the control of severe asthma. The remarkable variability observed in the individual responses to these novel biologic therapies further emphasizes the necessity of accurate asthma phenotyping, in order to achieve the best possible patient-focused management. In consideration of the frequently reported, only partial efficacy of the blockade of a single cytokine, the next research challenge might be represented by the opportunity of exploring, in carefully selected asthmatic subjects, the effects of different cocktails of biologics targeting the key pathogenic pathways underlying the various phenotypic subgroups of asthma. Of course, ongoing advances in our understanding of asthma pathobiology could make it possible, in the near future, to further extend the already promising scenario of anticytokine therapies for this sometimes hard-to-manage disease.
